# Thin Diamond Film on Silicon Substrates for Pressure Sensor Fabrication

**DOI:** 10.3390/ma13173697

**Published:** 2020-08-21

**Authors:** Stefano Salvatori, Sara Pettinato, Armando Piccardi, Vadim Sedov, Alexey Voronin, Victor Ralchenko

**Affiliations:** 1 Engineering Faculty, Università Niccolò Cusano, Via don Gnocchi 3, 00166 Rome, Italy; sara.pettinato@unicusano.it (S.P.); armando.piccardi@unicusano.it (A.P.); 2 CNR–IMM Institute for Microelectronics and Microsystems, Via del Fosso del Cavaliere 100, 00133 Rome, Italy; 3 Prokhorov General Physics Institute, Russian Academy of Sciences, Vavilov street 38, Moscow 119991, Russia; sedovvadim@yandex.ru (V.S.); vg_ralchenko@mail.ru (V.R.); 4 Research and Production Corporation “Istok”, Fryazino 141190, Russia; alexey-voronin@inbox.ru

**Keywords:** CVD diamond deposition, polycrystalline diamond films, high-pressure measurement, Fabry–Pérot cavity, harsh environment

## Abstract

Thin polycrystalline diamond films chemically vapor deposited on thinned silicon substrates were used as membranes for pressure sensor fabrication by means of selective chemical etching of silicon. The sensing element is based on a simple low-finesse Fabry–Pérot (FP) interferometer. The FP cavity is defined by the end-face of a single mode fiber and the diamond diaphragm surface. Hence, pressure is evaluated by measuring the cavity length by an optoelectronic system coupled to the single mode fiber. Exploiting the excellent properties of Chemical Vapor Deposition (CVD) diamond, in terms of high hardness, low thermal expansion, and ultra-high thermal conductivity, the realized sensors have been characterized up to 16.5 MPa at room temperature. Preliminary characterizations demonstrate the feasibility of such diamond-on-Si membrane structure for pressure transduction. The proposed sensing system represents a valid alternative to conventional solutions, overcoming the drawback related to electromagnetic interference on the acquired weak signals generated by standard piezoelectric sensors.

## 1. Introduction

Diamond represents an attractive material in different fields and applications due to its peculiar chemical and physical characteristics. Despite its excellent mechanical and tribological properties, applications of natural diamond have been limited by the high cost due to the scarcity of useful size for gems, as well as the necessary mechanical post-treatments. The progress of research in high-quality diamond synthesis by means of Chemical Vapor Deposition (CVD) techniques has promoted the use of diamond in different engineering tasks, both because of the excellent properties of grown material and because of the reduction in diamond production costs [1]. Currently, CVD processes allow the use of synthetic diamond for coating and the fabrication of thin and thick free-standing films, wafers, and windows with a quality tailored for the specific applications.

Modern CVD techniques are used for growth of single crystal (SC) and polycrystalline diamond (PCD) films. SC diamond of very high purity can be obtained having an electronic grade quality, while PCD display interesting characteristics in terms of mechanical, electronic, and optical properties combined with large film area [1,2]. First of all, being a wide bandgap semiconductor (5.47 eV at room temperature), it offers a large number of advantages over other electronic materials, resulting particularly attractive for the realization of electronic devices used at temperatures in excess of 300 °C [3]. For its high atomic volume density in lattice (10^23^ atoms/cm^3^) and its radiation hardness, it represents a key solution when used as an active material for the fabrication of miniaturized detectors for impinging charged particles [4,5,6,7,8,9], as well as for soft X-rays [10,11,12] and UV [13,14,15] detection. Moreover, overcoming the lack of efficient n-doping effect at room temperature of diamond, hence, the fabrication of p-n junction-based devices, post-treatments based on either laser- or ion beam-processing are able to induce a local transformation of diamond into graphite, representing a powerful methodology for the development of novel all-carbon devices for optoelectronics and photonics applications [16,17,18,19,20,21].

The high elasticity of diamond makes it attractive for both microelectromechanical systems and capacitive micromachined ultrasonic transducers fabrication [22,23,24]. In addition, polycrystalline and single-crystal diamond membranes have been proposed as masks for X-ray lithography [25], in nanotechnology [26], for the fabrication of hybrid devices in integrated photonics [27], as dielectric for capacitor used at high temperatures [28], as vacuum windows for ion microbeam transmission [29], and as super-thin active material for radiation detection [30].

Diamond films with thicknesses ranging from a few microns to hundreds of microns are usually grown on silicon substrates by the microwave plasma-assisted CVD technique (MPCVD). Homogeneous diamond films with a low defect density are required in several applications. A fundamental step is nucleation enhancement, because by this phase, it is possible to obtain different configurations with different mechanical, thermal, and electrical characteristics [31,32]. Over the years, many efforts have been made to improve nucleation efficiency with respect to the orientation of the nucleated diamond grains. The use of a porous silicon layer demonstrated its effectiveness, providing nucleation sites by trapping the diamond seeds in its pores, thus, obtaining a good nucleation density and uniform diamond deposition [33]. Pre-treatment of substrates prior to diamond deposition significantly improves the nucleation density also for crystalline silicon. For example, dry polishing allows a uniformly sown diamond layer to be obtained. In particular, crystalline silicon wafers are seeded with nanodiamond (ND) particles using an ultrasonic bath in water-based suspension of ND powder. Such a pre-treatment provides homogeneous seeding with a nucleation density over 10^9^ cm^−2^ [34].

It is worth pointing out that CVD diamond, in single-, poly-, micro-, or nano-crystalline forms, displays physical properties comparable to those of natural diamond [2,3,35]. Hence, it is particularly interesting in several application fields due to: the high value of its Young’s module (1143 GPa); a low thermal expansion coefficient (1.0–1.5 ppm/°C); a high thermal conductivity (2200 W m^−1^ K^−1^) compared to other materials; a high melting temperature (1700 °C in vacuum); a moderate refractive index value (2.4). Moreover, the abovementioned properties, combined with chemical stability and high hardness, make diamond particularly suitable when used in harsh environments.

In this context, pressure has always been one of the most important and critical parameters to be measured in various fields, including automotive, industrial, biomedical, and aerospace [36]. Nowadays, equipment able to measure pressure and pressure variations, even in aggressive media, is required. For this reason, for several years, diamond has been considered a suitable material for pressure sensors used in harsh and aggressive environments. Indeed, due to aforementioned excellent mechanical characteristics, CVD diamond membranes are suitable for pressure sensors fabrication [37]. However, the most common diamond pressure sensors typically exploit piezoresistive properties of boron-doped material [38]. Conversely, optic sensors based on Fabry–Pérot interferometric measurements are relatively new in the field of diamond-based sensing devices, although they intrinsically show clear advantages of miniaturization, high sensitivity, chemical inertness, high operating temperatures, and especially, immunity to electromagnetic interference [39]. Exploiting the mentioned outstanding properties of diamond, as well as its biocompatibility, diamond films grown on silicon substrate have been used as reflective layers for fiber-optic displacement sensors, showing their performance in the range of 0–600 μm [40]. In addition, recent works report novel application of thin nitrogen-doped and boron-doped nanocrystalline diamond films on silicon as reflective surfaces in an interferometric sensor dedicated to measuring refractive indices of liquids [41]. Moreover, it is worth citing that optical interferometry, successfully applied to in situ monitoring of electropolymerization of melamine at the boron-doped nanocrystalline diamond electrode surface, represents a powerful technique for interactions occurring on the surface of the electrode during electrochemical reactions [42].

In this work, we show the design and realization of pressure sensors based on thin diamond diaphragms. Polycrystalline CVD diamond membranes with a thickness of the order of 6 µm deposited on a silicon substrate have been realized by selective etching of silicon. The obtained free-standing diamond film represents the pressure sensing diaphragm. A fiber-coupled optoelectronic system was used to measure the diaphragm deflection as a function of the pressure difference between the two faces of the membrane itself. Assuming, for diamond, the mechanical properties reported in the literature, silicon hole diameter and diamond film thickness have been chosen to easily detect a diaphragm deflection from tens of nm to tens of µm, as induced by an inlet pressure as low as a few bars. Fabrication processes have been performed in batches by growing PCD by means of the MPCVD technique, using conventional deep reactive ion etching semiconductor processes and laser-assisted lithography of the metal mask. Sensor fabrication steps are described in detail and preliminary experimental results under a pressure difference up to 16.5 MPa are illustrated in the following sections.

## 2. Materials and Methods

### 2.1. Diamond Membranes Fabrication

**Figure 1:**
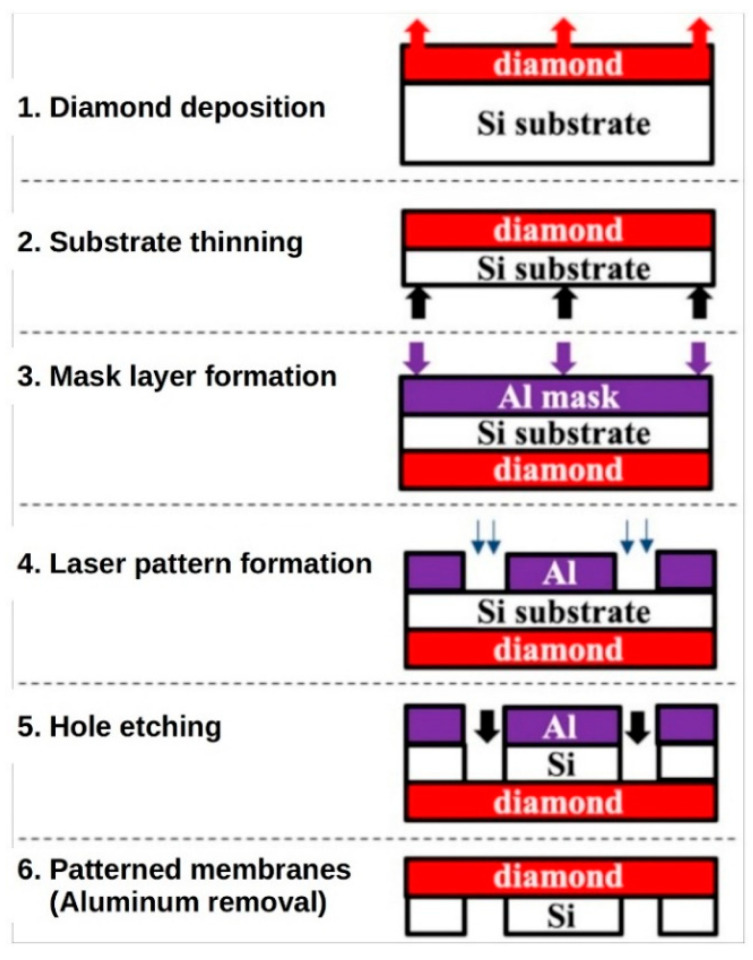
Scheme for the polycrystalline diamond membranes fabrication: (1) CVD growth of PCD films on Si substrate; (2) Thinning of the Si substrate with inductively coupled plasma etching; (3) Deposition of the thin aluminum mask; (4) Formation of windows in the Al mask using the excimer laser; (5) Local etching of Si substrate with the inductively coupled plasma etching technique; (6) Removal of the residual Al layer.

The diamond membranes were prepared using a six step procedure, previously reported by Sedov et al. [34], which is shown schematically in Figure 1.

First, the diamond film was grown by MPCVD on a Si substrate (step 1). Then, the Si substrate was thinned to a thickness of 120 µm (step 2) and an aluminum mask was deposited on the bottom side of the substrate (step 3). Next, circular windows in the metal mask were formed by laser ablation (step 4). The Si was selectively removed by plasma etching though the windows (step 5) to obtain an array of the diamond membranes supported on the Si substrate. Finally, the metal layer was removed by means of chemical wet etching (step 6).

A mirror-polished, 400 µm thick and 2-inch in diameter, single crystal (111)-oriented silicon wafer was used as substrate. To guarantee uniform deposition as well as full coverage of the substrate surface, the Si substrate was preliminary seeded with ND powder with an average particle size of about 5 nm (Daicel Corp., Osaka, Japan) to provide diamond nucleation centers. The seeding procedure was performed by immersing the substrate for 10 min in an ultrasonic bath with a water-based suspension of ND particles. The PCD film was deposited in CH_4_/H_2_ gas mixture on the pre-treated substrate by the MPCVD technique using an ARDIS-100 system (2.45 GHz, 5 kW, Optosystems Ltd., Troitsk, Russia) at the following process parameters: methane content of 6%, total gas flow of 500 sccm, gas pressure of 55 Torr and microwave power of 5.0 kW. The substrate temperature was maintained at ≈800 °C as measured with a two-color pyrometer Micron M770 (Mikron Infrared Inc., Oakland, CA, USA).

The growth rate of diamond was measured in situ with a laser interferometry technique [43,44]. The laser beam (λ = 650 nm) was directed almost perpendicularly to the growing surface through the top quartz window of the CVD reactor. The reflected beam was collected and directed to the optical spectrometer Ocean Optics 4000 through the same window. The thickness of the film increased by Δ*h* = λ/2*n* for one period of the reflected beam intensity oscillations, where *n* = 2.4 is refraction index of diamond (the increment Δ*h* is 134.5 nm). A growth rate of about 1.2 μm/h was determined for the early stage of the growth process (for the first 100 min).

**Figure 2:**
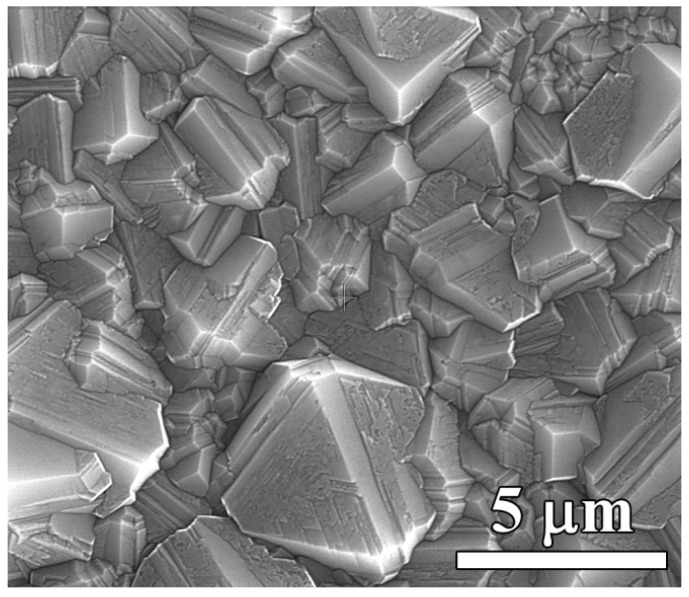
SEM image of the 5.9 μm thick polycrystalline diamond film grown on silicon.

After 5 h of CVD growth, a uniform polycrystalline diamond film was produced consisting of randomly oriented well-faceted grains with average size of ~4 μm when viewed on the growth side (Figure 2), as revealed with scanning electron microscopy (SEM) (TESCAN MIRA3 instrument, TESCAN ORSAY HOLDING a.s., Kohoutovice, Czech Republic). The film thickness evaluated from the weight gain of the sample was about 5.9 μm.

The substrate back side then was homogeneously thinned down to a thickness of ~120 µm using inductively coupled plasma etching (ICP) using a PLASMA TM5 system (NIITM, Moscow, Russia) operating at 13.56 MHz with an etch rate of ≈5 μm/min. A bi-layer metal mask consisting of a 100 nm thick Ti interlayer and 2 μm thick Al top layer was deposited on the Si substrate using electron beam physical vapor deposition technique (Evatec BAK 761, Evatec AG, Trübbach, Switzerland). The titanium interlayer promoted a better adhesion of the aluminum film to the Si. The windows in the mask were opened by ablation with a KrF excimer laser CL-7100 (Optosystems Ltd., Troitsk, Russia), wavelength λ = 248 nm, pulse duration τ = 20 ns, and repetition rate f = 50 Hz.

**Figure 3:**
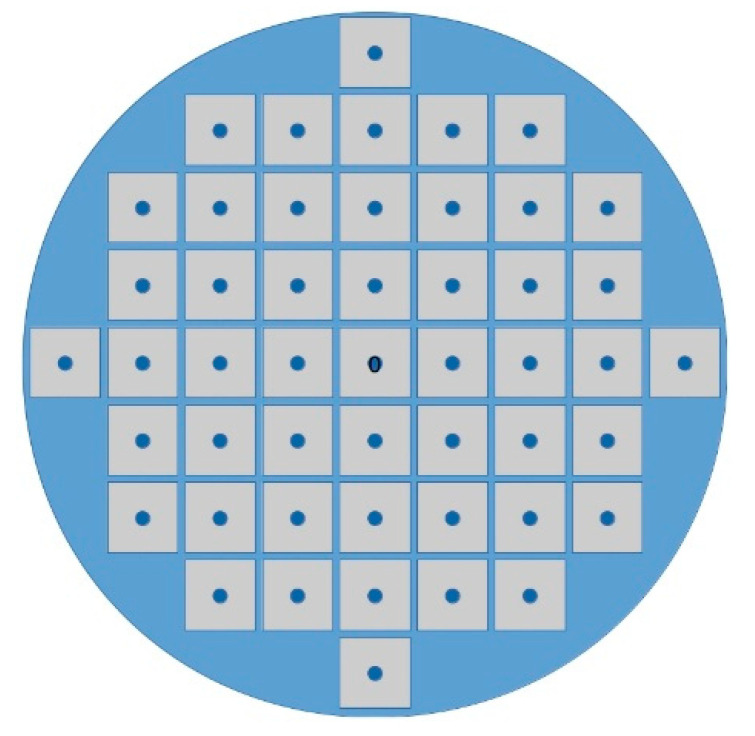
Schematic of the 5 × 5 mm^2^ sensing elements realized on the 2-inch silicon wafer. After slicing, about 50 different sensing elements were produced.

The sample was irradiated through circular holes of 3 to 8 mm in diameter in a tantalum mask using an optical projection scheme to transfer the hole image to the sample surface with de-magnification of a factor 20, so the diameter of holes obtained in the Ti/Al mask was between 150 and 400 μm. At the laser fluence of 1.5 J/cm^2^ (it exceeded the ablation threshold both for aluminum and titanium), 20 pulses were enough to completely remove the Ti/Al metallization in a given spot. The distance between two nearest-neighbor holes was 5 mm, resulting in about 5 × 5 mm^2^ the dimension of each sensor as schematically depicted in Figure 3.

ICP etching was used to selectively remove silicon from substrate areas unprotected by the Al-Ti mask. The plasma etching of the Al layer was negligible, and the etching automatically stopped when it reached the diamond/Si interface. The etching process was periodically interrupted to monitor the depth of holes with a Dektak 150 profilometer (Veeco Instruments Inc, San Jose, CA, USA).

**Figure 4:**
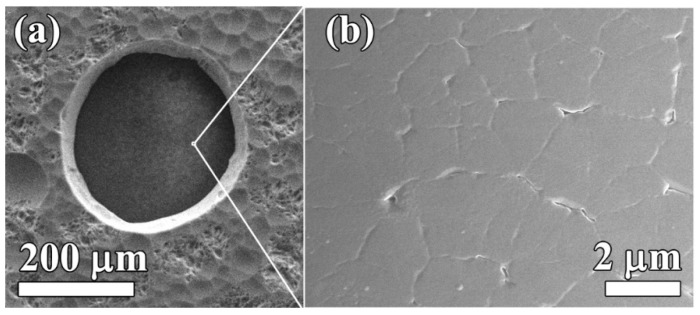
SEM images of a diamond membrane of viewed through a hole in thinned Si substrate (**a**), and the smooth diamond nucleation surface at higher magnification (**b**).

SEM images of the nucleation side of a membrane are displayed in Figure 4. When viewed on the holes in Si from the bottom side (Figure 4a), sidewalls with a tilt angle in the range of 7–10° are observed. As discussed below, such a geometry does not affect sensor functionality. The inspection at higher magnification of the nucleation side of the diamond film surface on the rear side of the membranes revealed it to be completely unaffected by the plasma etching procedure (Figure 4b). The diamond surface looks smooth and quite different from the morphology of the growth side (compared to Figure 2). The flat grains are bounded by narrow grooves formed between growing grains. When the faceted neighbor grains coalesce, they shadow the substrate preventing diamond from growing in the small region between facets, i.e., a gap forms there [45]. The grains develop to a larger size with an increase in the film thickness, thus, the PCD is an inherently gradient material with properties smoothly varied across the thickness.

The roughness of the PCD membrane was measured on both sides with the NewView5000 (ZYGO Corp., Middlefield, CT, USA) optical profilometer. A root mean square roughness *R_rms_* of 160 and 15 nm was measured for the coarse-grain growth surface and the nucleation surface, respectively.

**Figure 5:**
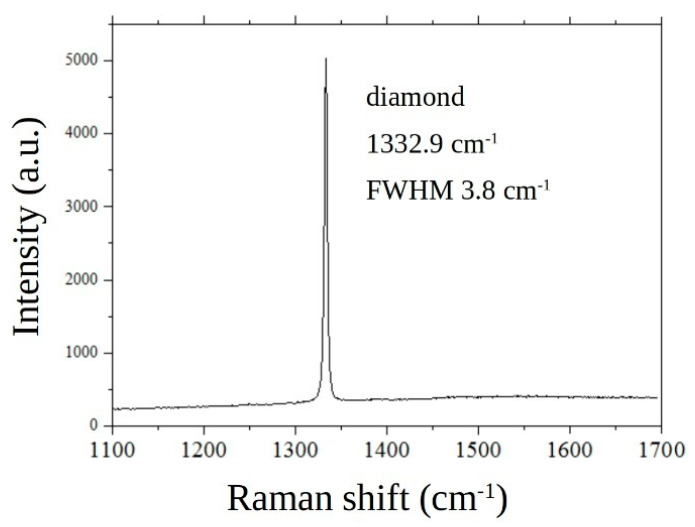
Raman spectrum taken on the growth side of a membrane. The sharp peak at 1332.9 cm^−1^ is associated to diamond.

The phase composition of the obtained film was analyzed at room temperature with micro-Raman spectroscopy using a LABRAM HR-800 spectrometer (Horiba, France) equipped with a diode-pumped solid-state laser (λ = 473 nm). The spectrometer operated in a confocal mode, while the laser beam was focused on a spot of about 1 μm in diameter on the sample surface. Figure 5 shows a typical Raman spectrum of the diamond film taken on the growth side. The only feature in the spectrum is the sharp 1st order diamond Raman peak at 1332.9 cm^−1^ with a full width at half maximum (FWHM) of 3.8 cm^−1^. No non-diamond phases like amorphous graphite or trans-polyacetylene inclusions [35] are revealed, evidencing a high-quality diamond material.

### 2.2. Pressure Sensor Structure and Measurement Set-up

**Figure 6:**
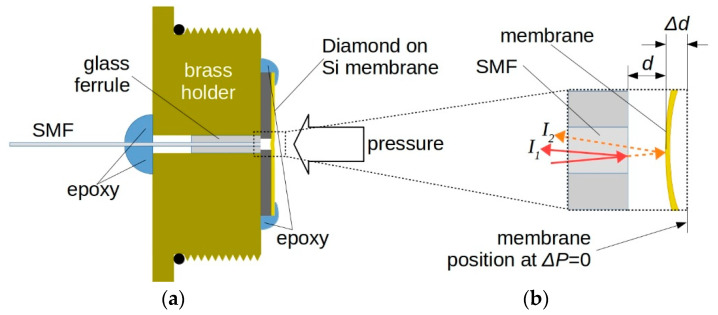
(**a**) Schematic of the sensor structure. SMF end-face and diaphragm represent the Fabry–Pérot cavity. (**b**) Interference signal is generated by the reflected signal at the fiber end (light intensity *I*_1_) and diamond membrane (light intensity *I*_2_). The cavity length *d* depends on the pressure difference Δ*P* on the two membrane surfaces, creating a displacement Δ*d* from the position when the same ambient pressure is applied.

Figure 6a illustrates the sensor structure schematic. The diamond-on-Si sample is aligned to a single mode fiber (SMF). SMF end-face and the inner surface of the diamond diaphragm realize a Fabry–Perot optical cavity. The fiber has a mode field diameter of about 10 μm at 1550 nm, and a nominal cladding and coating diameter of 125 and 245 μm, respectively (SMPF0215-FC, Thorlabs Inc., Newton, NJ, USA). As depicted in Figure 6b, laser light propagating into the SMF is partially reflected at the SMF facet (continuous arrow, *I*_1_), whereas light transmitted at the fiber output is reflected by the diamond film nucleation side (dotted arrow, *I*_2_). Hence, the reflected beam propagates back into the fiber and generates an interference pattern dependent on the cavity length, which, in turn, is a function of the pressure inducing diamond membrane deformation.

**Figure 7:**
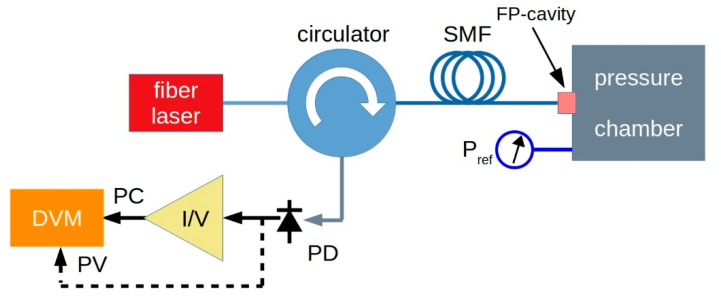
Schematic of the experimental set-up. An IR fiber laser is coupled to the FP cavity by means of a circulator. The interference signal is detected by an InGaAs photodiode (PD). Either photovoltage (PV) or photocurrent (PC) signal are acquired as a function of the pressure by a computer-controlled digital voltmeter (DVM). For photocurrent acquisition, a current-to-voltage (I/V) photodiode amplifier is inserted.

The schematic of the experimental arrangement used for measuring diamond membrane deflection is shown in Figure 7. An infrared (IR) fiber-coupled laser diode operating at 1550 nm (LPSC-1550-FC, Thorlabs Inc., Newton, NJ, USA) was used as the light source. A 3-port optical circulator (6015-3, Thorlabs Inc., Newton, NJ, USA) allowed the illumination of the membrane (FP cavity), whereas an InGaAs IR photodiode (PD, FGA01FC, Thorlabs Inc., Newton, NJ, USA) collected interference light and generated an electric signal. A transimpedance photodiode amplifier PDA200C (Thorlabs Inc., Newton, NJ, USA) coupled to a digital voltmeter (DVM, Keithley 2700 series, Tektronix Inc., Beaverton, OR, USA) was used for photocurrent (PC) signal conversion and computer-controlled voltage signal acquisition. In addition, photovoltage (PV) recording of the signal directly generated by IR PD was also performed by means of the DVM, hence, excluding the transimpedance amplifier conversion.

**Figure 8:**
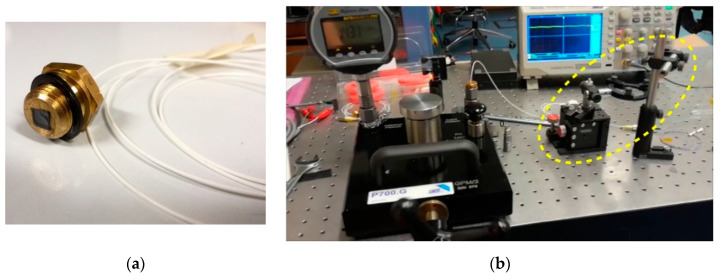
(**a**) Picture of a diamond-on-silicon membrane mounted on the brass holder and coupled to the SMF. (**b**) Picture of the experimental set-up used during high-pressure membrane characterization. On the left, the hydraulic test pump with the digital test gauge used for reference. On the right, the dotted circle indicates the micrometric x–y stage used for preliminary diamond-on-Si assembly on brass holder (see text).

The beam coming from the fiber needs to be aligned with the center of the membrane in order to maximize the system sensitivity. The SMF, terminated with a glass ferrule having a diameter of 1.800 ± 0.005 mm, was preliminary inserted into a though-pass hole of the brass holder. The glass ferrule end was precisely inserted up to the holder surface where the diamond-on-Si die is placed. Finally, SMF was fixed with epoxy. Rather than using a completely mechanical system for alignment, which could result complex and subject to relevant error, we employed an optical method, mounting the brass holder on a micrometric x–y stage to adjust the relative position with respect to the membrane, and used the same principle of the interference-detecting set-up of Figure 7. Once mounted on the fiber end on the brass adapter, the membrane was placed in close proximity of the fiber. The diamond-on-Si die was maintained stable with a simple weak-vacuum holding system. The reflected interference beam was detected as a function of the relative x–y position by means of the IR detector directly connected to a digital oscilloscope (PV-mode). Until the ray beam is reflected by silicon substrate, the obtained signal results stable over time. Conversely, when light impinges the membrane, oscillations induced both by vacuum pump and micrometric stage resulted in a time varying signal. By adjusting the fiber position to maximize the alternated root mean squared signal component, we ensured the beam was impinging on the most sensitive part of the membrane, i.e., its center. Then, in such a position, the 5 × 5 mm^2^ die was finally glued on the brass support. Figure 8a shows an example of a diamond-on-Si sensing element mounted on the brass holder. It is worth observing that, as the diamond diaphragm is illuminated on nucleation side, the extremely low value found for roughness (see Section 2.1) guarantees a lower loss of reflected light due to unavoidable scattering of impinging radiation, hence, a better sensor sensitivity.

For sensor characterization as a function of pressure on the membrane, the sensing element was inserted in a high-pressure chamber. Stationary pressure on the sensor membrane was regulated with a hydraulic table top test pump (P700.G, Sika, Kaufungen, Germany). At the reference port, inlet pressure was measured with a BetaGauge PI PRO Digital Test Gauge (Martel Electronics Corp., Derry, NH, USA). A picture of the implemented set-up is reported in Figure 8b, where the micrometric x–y stage used for cavity alignment is also shown (see dotted circle).

## 3. Results and Discussion

As described in the previous section, the SMF used as the input–output fiber and the diamond diaphragm used as the reflector form an air-filled gap acting as a low-finesse FP cavity. Analysis is largely simplified if the FP cavity can be assumed as a two-wave interferometer, i.e., neglecting the contribution of the outer surface of the diaphragm (diamond film growth side). Considering the interference under plane-wave approximation, each coherent light beam can be expressed in terms of its associated electric field *U*:

(1) $$U_{i}=\;A_{i}e^{j\varphi_{i}}\;;\;i=1,\;2$$

where *A_i_* and *ϕ_i_* are the wave amplitude and phase, respectively, and where *I* = *U*^2^ is the light intensity. Then, the superposition of the two plane-waves gives [46]

(2) $$I={\left|U_{1}+U_{2}\right|}^{2}=\;A_{1}^{2}+\;A_{2}^{2}+2A_{1}A_{2}cos\left(\varphi_{1}-\varphi_{2}\right)$$

which represents the light intensity detected by the IR photodiode. If *A*_1_ beam is considered as reference, *ϕ*_1_ = 0 and *ϕ*_2_ = 2*d*(2*π/λ*), with *λ* the wavelength of light and *d* the pressure dependent FP cavity length, *d = d*_0_ *±* Δ*d*, where Δ*d* is the displacement from the initial position *d*_0_ when the pressure on the two membrane surfaces is the same (ambient pressure). Then, previous equation becomes

(3) $$I\left(\lambda ,d\right)=\;A_{1}^{2}+\;A_{2}^{2}+2A_{1}A_{2}cos\left(\frac{4\pi d}{\lambda }\right)$$

giving the relationship between SMF diamond diaphragm gap displacement and power loss of detected light. The PD output photocurrent signal (*I_ph_*) has, then, a sinusoidal behavior as a function of the diaphragm displacement, in which the period depends on the *d/λ* ratio. Peak-to-peak amplitude and offset of the signal will depend on the relative intensities of *A*_1_ and *A*_2_. A fringe period corresponds to a phase change of 360° in the sensing reflection, i.e., 775 nm for the 1.55 µm laser source used during characterization.

The diamond diaphragm is compressed towards the fiber end-face by the applied pressure and the cavity length reduces by Δ*d* at the center (see Figure 6b). As the optical fiber core diameter is much lower than the diaphragm diameter, optical interference is mainly induced by the center deflection of the membrane. Hence, the observed 7.7° tilt of etched silicon, aforementioned in Section 2.1., does not influence the output signal. Conversely, the alignment between fiber core and silicon hole ensures optimal collection of reflected light. Assuming a uniform thickness *t* for the membrane, under a uniformly distributed pressure difference Δ*P* between the two membrane surfaces, the maximum deflection of a circular diaphragm of radius *a*, occurring at the center position, is given by [47]

(4) $$\Delta d=\frac{a^{4}}{64D}\Delta P$$

where

(5) $$D=\frac{Et^{3}}{12\left(1-\nu^{2}\right)}$$

is the flexural rigidity of the plate, whereas *E* and *ν* are Young’s modulus and Poisson’s ratio of the diaphragm, respectively.

**Figure 9:**
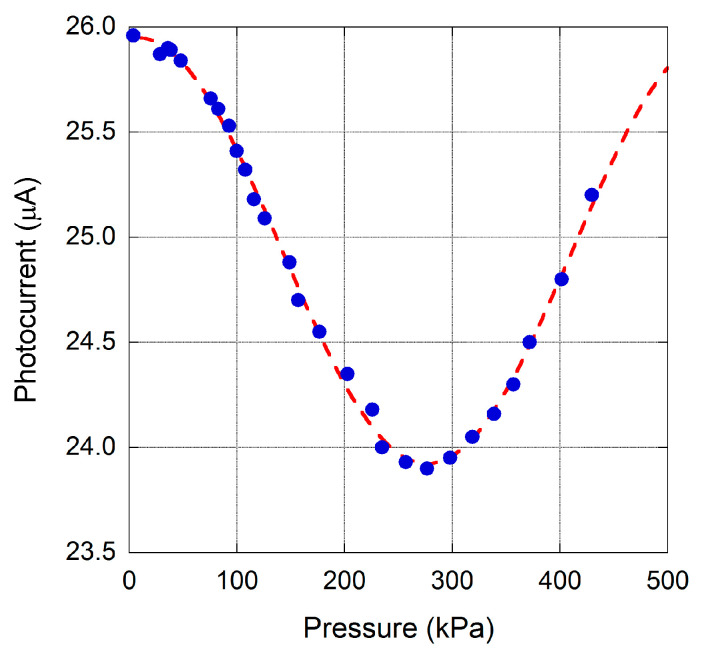
Photocurrent signal acquired for a membrane of 360 µm diameter for pressure up to 430 kPa. The dotted line represents best fit of experimental data, according to Equation (3).

Preliminary characterization of a sample has been conducted by means of the above described experimental set-up. Figure 9 reports photocurrent data, which are proportional to PD impinging light power and acquired for a sensing element up to a pressure difference Δ*P* of 430 kPa. A membrane diameter of about 360 ± 20 µm was evaluated with optical microscope.

Best fit of experimental data according to the sinusoidal expected behavior given by Equation (3) allows the estimation of the fringe-period of output signal. In particular, a 360° of phase shift between *A*_1_ and *A*_2_ beams is evaluated for a pressure difference of 540 ± 8 kPa, which corresponds to Δ*d* = 775 nm. It is worth noting that, assuming a Poisson’s ratio of 0.07 and a nominal Young’s modulus value around 1100 GPa for diamond [48], from Equations (4) and (5), an effective thickness *t* ≈ 5 µm is calculated for the results depicted in Figure 9, in good agreement with the value of 5.9 μm estimated during diamond film growth.

**Figure 10:**
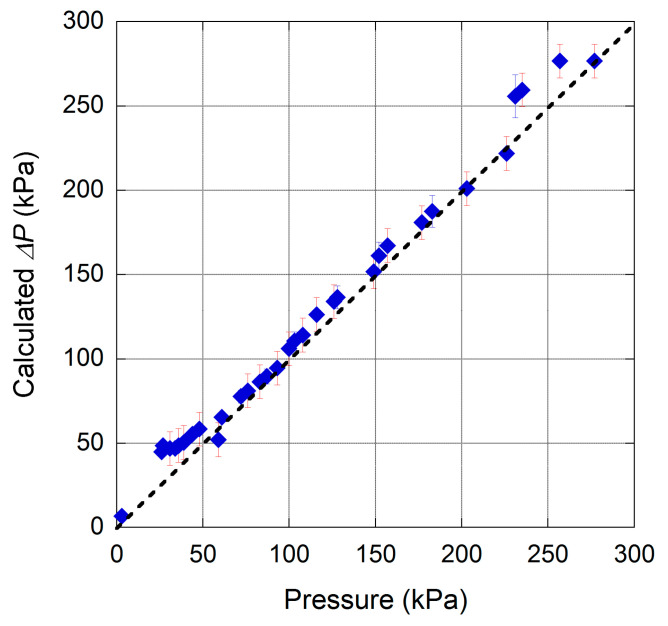
Sensor repeatability has been evaluated under several 0–300 kPa cycles. Best fit of experimental data reported in Figure 9, used to calculate the sensor transfer characteristics, allowed estimation of the inlet pressure difference Δ*P*.

The parameters derived by best fit of experimental data have been used to calculate the Δ*P*–*I_ph_* sensor transfer characteristic. Then, sensor functionality, in terms of repeatability, has been evaluated with three cycles of pressure changes. In particular, the pressure was increased and decreased in the range 0–300 kPa. It is worth to mention that hysteresis effects have not been observed during such a characterization. As illustrated in Figure 10, a fairly good linearity is observed in the 40–230 kPa range, with an absolute error lower than 10 kPa.

**Figure 11:**
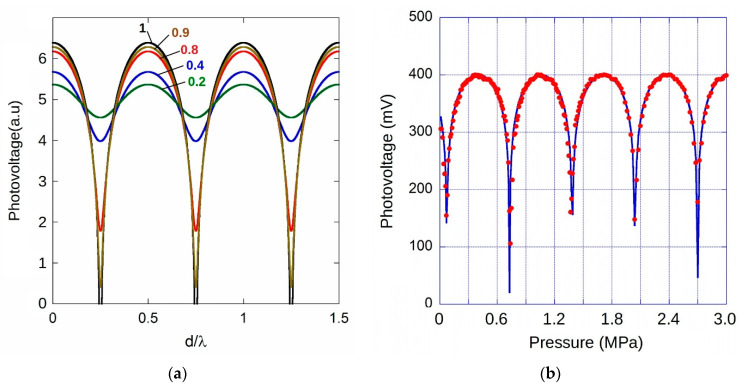
(**a**) Simulated photovoltage output signal behavior of PD as a function of the FP cavity length normalized to the wavelength of impinging light for different *A*_2_/*A*_1_ intensity ratio of reflected beams. (**b**) Photovoltage output signal of PD as a function of the pressure difference for a 380 µm in diameter PCD membrane.

**Figure 12:**
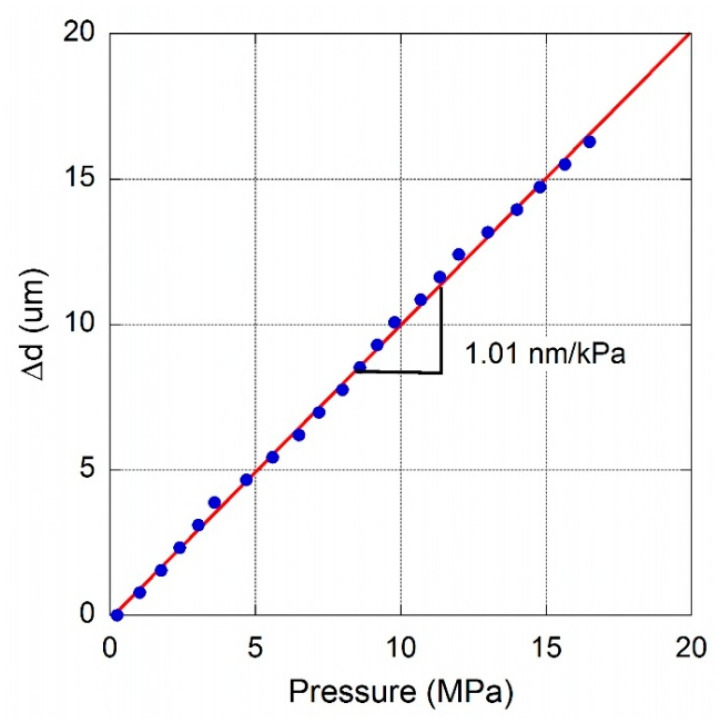
Membrane deflection as a function of pressure difference evaluated at minimum of signal of a measurement performed up to 16.5 MPa for a PCD membrane having a diameter of 380 µm.

It is worth observing the completely different behavior expected acquiring PD output signal in PV-mode. Figure 11a shows simulations of Equation (3) for different values of *A*_2_/*A*_1_ light intensities ratio. Due to the logarithmic nature of photodiode response on the light intensity, it is worth noting that for a *A*_2_/*A*_1_ ratio greater than 0.8, a steeper response would be observable in correspondence of destructive interference between beams. Conversely, for intensity ratio lower than 0.4, a “sinusoidal-like” behavior, with low dynamics, would be found. Figure 11b shows the experimental result for a sample with a membrane diameter of 380 ± 20 µm in the 0–3 MPa pressure range. Acquired signal displays the expected interference behavior with a periodicity corresponding to a *λ*/2 diaphragm shift, i.e., 775 nm. The steeped response observed when signal drops states a good collection of both the two reflected and interfering light beams. Blue line represents best fit result according to the logarithm of Equation (3), highlighting the good alignment performed in this case. Measurements have been repeated up to more than 16.5 MPa and Figure 12 summarizes data points observed at signal minimum (=*λ*/2 of membrane deformation). A fairly good linearity is found in the investigated range, with a slope of 1.01 ± 0.01 nm/kPa. Assuming for Poisson’s ratio and Young’s modulus the same values used for the previous sensing element (same batch), from Equations (4) and (5), an effective thickness t ≈ 6 µm is estimated for results depicted in Figure 12. Although a deeper investigation on long term stability of membranes subjected to hundreds or thousands of cycles of pressure changes would be necessary from a practical point of view, for the investigated samples, optical microscopy did not reveal any modification of diaphragm structure such as deformation or cracks, highlighting the good quality of the PCD films.

## 4. Conclusions

The operation of a simple, low-finesse, extrinsic FP sensor based on diamond on silicon membranes has been reported. The adopted six-step diamond-on-Si diaphragm fabrication procedure allowed the production of high-quality thin polycrystalline diamond films which display an extremely good mechanical elasticity with a Young’s modulus and Poisson’s ratio values in agreement with those reported in the literature. Experimental test data were obtained for membranes 5.9 µm thick and have demonstrated the feasibility of the proposed sensor structure, validating the presence of a true FP interferometer in agreement with the expected behavior. Analyzed sensing elements displayed a good linearity in the investigated high-pressure ranges; more in detail, in the ranges 40–230 kPa and 0–16.5 MPa for membranes with diameter of 360 and 380 µm, respectively. In the first case, an error of less than 10 kPa was found, while in the second case, a sensitivity of about 1 nm/kPa was evaluated. Moreover, it is worth noting that assuming a Poisson ratio of 0.07 and a Young’s module nominal value around 1100 GPa for diamond, the calculated diaphragm thicknesses are in good agreement with the 5.9 µm value evaluated from the weight gain of the 5-inch sample. Such a result confirms the good quality of the deposit also in terms of thickness uniformity over the 5-inch silicon substrate, allowing the realization of about 50 different sensing elements with the same technological processing. Membranes geometry was defined by means of selective silicon etching, implementing a laser-assisted metal mask lithography. The method would also allow the definition of different geometries not limited to the circular one adopted for the present work. Preliminary characterization demonstrates the feasibility of the optical system for pressure measurement. It is worth noting that for the proposed structure, the only sensing element immersed in the pressure chamber is diamond, allowing the sensor to work in a harsh environment in which the material demonstrates its chemical inertness. In addition, the proposed optical sensing system would represent a good choice in environments where noise introduced by electromagnetic interference makes difficult the use of piezoresistive sensors.
